# Digging out MDIG from the mess of H3K9me3, OTX2 and MYC signaling in human cancers

**DOI:** 10.7150/ijbs.92589

**Published:** 2024-01-21

**Authors:** Ziwei Wang, Chitra Thakur, Akimasa Seno, Fei Chen

**Affiliations:** 1 Stony Brook Cancer Center, and Department of Pathology, Renaissance School of Medicine, Stony Brook University, Lauterbur Drive, Stony Brook, NY 11794, USA; 2 Faculty of Engineering, Graduate School of Interdisciplinary Science and Engineering in Health Systems, Okayama University, Okayama 700-8530, Japan, and R&D Center, Katayama Chemicals Ind., Co. Ltd, Ina, Minoh, Osaka, 562-0015, Japan

As the largest organ in the body of mammals, the liver possesses an exceptional capacity to restore its function following acute injury or toxic damage through regeneration. A subpopulation of hepatocytes with stemness properties is arguably the cellular source in regeneration, maintaining tissue homeostasis. Under certain circumstances, regeneration is accompanied by an overactivation of hepatic stellate cells, leading to liver fibrosis or cirrhosis 1, which is a common etiological factor for the development of hepatocellular carcinoma (HCC). It is generally accepted that an intricate signaling network is essential for collective cellular behaviors during regeneration. However, a conserved repertoire of genes controlling this process remains to be fully established.

In a recent issue of Signal Transduction and Targeted Therapy (STTT), Du *et al.* employed liver-specific gene editing technique to dissect the stepwise emergence of the MDIG-mediated signaling network in experimental liver regeneration in mice 2. MDIG has been identified as an oncogenic protein containing JmjC domain, with bifunctional activities of histidine hydroxylase and lysine-specific demethylase 3. In line with the growing evidence of MDIG's demethylase-like activity, the authors revealed an inverse temporal relationship between MDIG expression and H3K9me3 demethylation in the liver following partial hepatectomy (PH) or CCl_4_ treatment. As expected, PH or CCl_4_ challenge induced a rapid increase in MDIG protein and a concomitant decrease in H3K9me3 in the liver, accompanied by a notable recovery of liver mass in wild-type mice. In contrast, in mice with liver-specific deletion of MDIG, the level of H3K9me3 remained high, and the recovery of liver mass was substantially delayed. These findings indicate the demethylase-like activity of MDIG on H3K9me3 and underscore the importance of MDIG in the growth or proliferation of hepatic cells, most likely, the hepatocytes. To dissect how the antagonism of MDIG on H3K9me3 contributes to liver regeneration, the authors further explored chromatin profiling of the livers of WT and MDIG KO mice using ATAC-seq and gene specific ChIP-PCR. The results suggested that *MDIG* knockout weakened chromatin accessibility due to the enhanced enrichment of H3K9me3 at the promoter region of *OTX2*, a key gene regulating the proliferation and differentiation of neural cells. Additional biochemical tests confirmed the stepwise emergence of the signaling cascade initiated by MDIG in liver regeneration. In response to PH or CCl_4_ challenge, increased MDIG demethylated H3K9me3 on the locus of the OTX2 gene and many other genes, leading to an upregulation of OTX2. OTX2, in turn, governs the transcription of MYC, a critical effector of MDIG, ultimately resulting in enhanced liver regeneration characterized by increased expression of several cell cycle-regulating genes.

Currently, there is ongoing discussion about the role of MDIG as a histone demethylase. In human lung cancer, the cancer tissues display elevated levels of MDIG protein, coupled with reduced H3K9me3 compared to the case-matched normal lung 4. This notion is reinforced by our ChIP-seq analyses examining the global distribution of H3K9me3 in both the bronchial epithelial cell line BEAS-2B and the triple-negative breast cancer cell line MDA-MB-231 cells 5, 6 (Figure 1A). Notably, in both cells lines, the knockout of the MDIG gene results in a significant enhancement of H3K9me3. The data from Du *et al.* provide another line of evidence supporting the involvement of MDIG in histone demethylation, albeit indirectly 2.

OTX2 functions as an oncogenic transcription factor in certain cancer cells and stem cells 7. In human induced pluripotent stem cells (iPSCs), the OTX2 gene locus is featured with multiple enriched H3K4me3 and H3K36me3 peaks, but notably lacks the major repressive marker H3K9me3 (Figure 1B). Conversely, in well-differentiated BEAS-2B cells, OTX2 gene resides in a transcriptionally inactive region on chromosome 14. There are no detectable active transcriptional markers, such as H3K4me3, H3K4me1, H3K27Ac, and NRF2, at the OTX2 gene locus (unpublished). Deletion of MDIG leads to an increased enrichment of H3K9me3 at -1500bp, -800bp, -400bp, TSS, +500bp, and down-stream of human OTX2 gene (Figure 1B, right panel). This pattern aligns with the ChIP-PCR results reported by Du *et al*. 2 for the mouse OTX2 gene. All of these findings collectively emphasize the crucial role of the epigenetic status governed by MDIG in OTX2 gene expression and highlight the pro-proliferative nature of MDIG, OTX2 and MYC in cells, as concluded by Du *et al*. 2.

Liver regeneration and HCC share numerous common genetic and epigenetic signatures, with abnormal regeneration often precedes malignancy 8. It is, therefore, not surprising that an increase in MDIG expression has been observed in approximately 65% to 70% of human HCC cases 9, 10. The findings by Du *et al*. 2 undoubtedly mark a significant stride in the mechanistic comprehension of liver regeneration. However, these findings also give rise to new questions. Firstly, it remains unclear whether the results from animal studies can be extrapolated to human liver regeneration or HCC, as no studies have yet explored the role of MDIG in human liver regeneration. Secondly, MDIG exhibits seemingly opposite effects in the early phase (24-72h) and late phase (120-168h) of liver regeneration 2. Could this discrepancy be attributed to an over-compensating growth of hepatocytes in the later phase? Lastly, in human HCC, elevated levels of MDIG and MYC are associated with poorer survival, while high expression of OTX2 is a strong predictor for better patient survival (Figure 1C), indicating that it is highly unlikely that OTX2 can serve as an oncogenic factor in HCC. Unlike MDIG, which shows increased expression in most types of human cancers, OTX2 expression is nearly undetectable in 21 listed tumors, except testis cancer, in the TNMplotter database (Figure 1D). Furthermore, no significant correlation of MDIG and OTX2, neither positive nor negative, can be detected in a database containing 40,442 tumors and 15,648 normal tissues representing 22 tissue orgins. Thus, OTX2 may not be among the major MDIG target genes and may be less important in the initiation and progression of human cancers resulting from MDIG overexpression. In conclusion, additional research is warrented to underscore the oncogenic role of MDIG as well as OTX2 in human cancer.

## Figures and Tables

**Figure 1:**
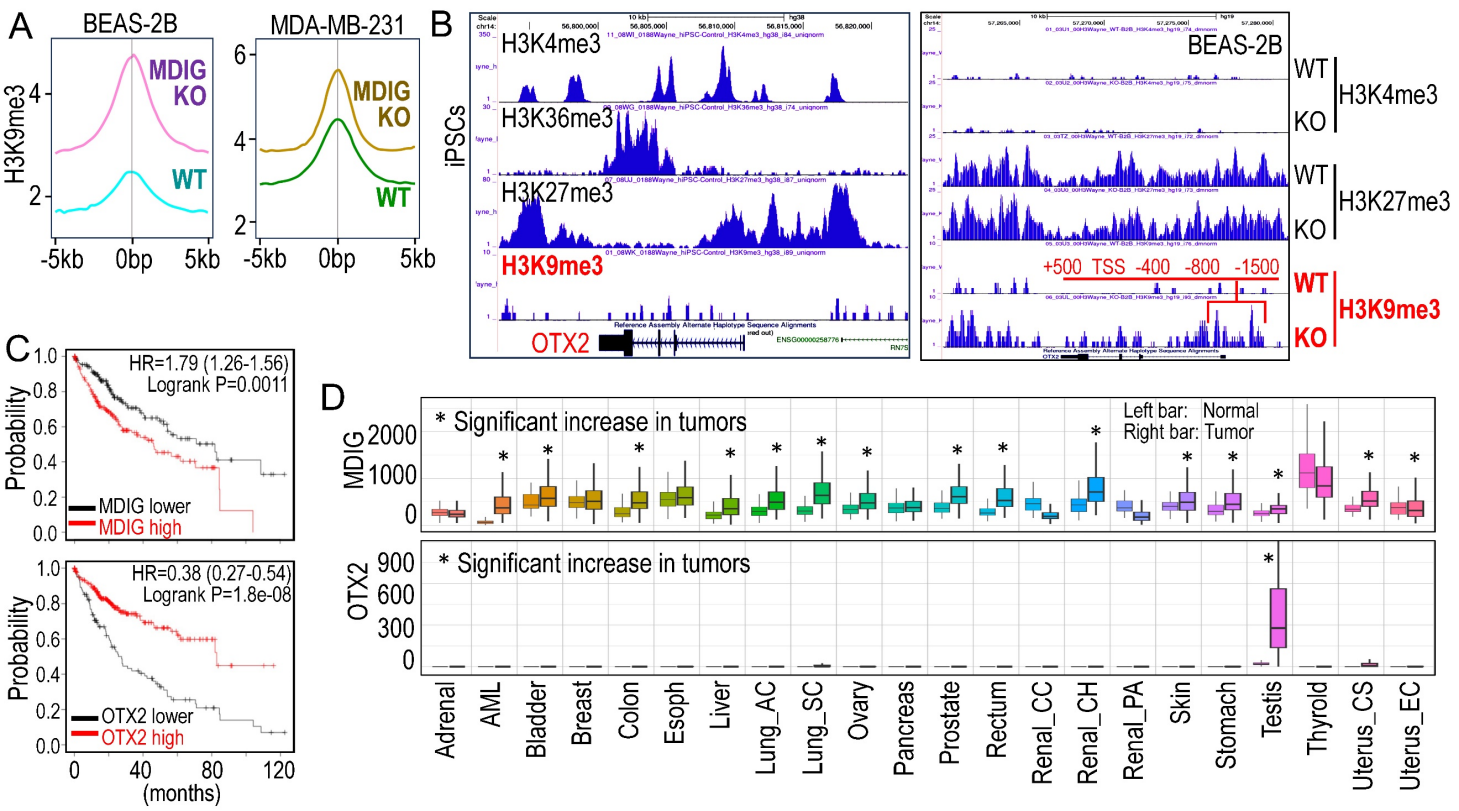
Antagonism of MDIG on histone H3 lysine 9 trimethylation (H3K9me3). **A.** ChIP-seq reveals that MDIG knockout enhances H3K9me3 enrichment on the genome of human bronchial epithelial cell line BEAS-2B cells (left) and human triple negative breast cancer cell line MDA-MB-231 cells (Right). **B.** OTX2 gene is enriched with peaks of active transcription markers (H3K4me3 and H3K36me3), but not repressive marker H3K9me3 in human induced pluripotent stem cells (iPSCs, Nips-B2). The bivalency of H3K27me3 and H3K4me3 indicates the poised status of OTX2 in iPSCs (left). Right panel: OTX2 gene is located in a transcriptionally inactive region on chromosome 14 featured with deminished H3K4me3 peaks in BEAS-2B cells. Knockout of MDIG (KO) enhanced the enrichment of H3K9me3 on OTX2 gene. Red lines and numbers indicate the positioning of H3K9me3 peaks in MDIG KO cells identified by ChIP-seq, which is similar to the findings by Du *et al.* in ChIP-PCR for the mouse OTX2 gene 2. **C.** Opposite effects of MDIG and OTX2 on HCC patient survival. High expression of MDIG predicts poorer survival (upper), whereas high expression of OTX2 predicts better survival of the patients with HCC (bottom). Data are derived from the Kaplan-Meier cancer patient survival and RNA-seq gene expression database by auto selection of the best cutoff values between the computed lower and upper quartiles. **D.** Significantly increased expression of MDIG in the indicated tumors relative to the case-matched normal tissues (upper), whereas except testis tumor, OTX2 is nearly undetectable in the indicated 21 types of tumors (bottom). Data are extrapolated from TNMplotter database using the relative expression values of MDIG and OTX2 mRNAs. (All data in this figure are from the authors' laboratory of this commentary).
